# Sub-2 Å Ewald curvature corrected structure of an AAV2 capsid variant

**DOI:** 10.1038/s41467-018-06076-6

**Published:** 2018-09-07

**Authors:** Yong Zi Tan, Sriram Aiyer, Mario Mietzsch, Joshua A. Hull, Robert McKenna, Joshua Grieger, R. Jude Samulski, Timothy S. Baker, Mavis Agbandje-McKenna, Dmitry Lyumkis

**Affiliations:** 1 The National Resource for Automated Molecular Microscopy, Simons Electron Microscopy Center, New York Structural Biology Center, 89 Convent Ave, New York, NY 10027 USA; 2 Department of Biochemistry and Molecular Biophysics, Columbia University, New York, NY 10032 USA; 3 Laboratory of Genetics and Helmsley Center for Genomic Medicine, The Salk Institute for Biological Studies, La Jolla, CA 92037 USA; 4 Department of Biochemistry and Molecular Biology, Center for Structural Biology, The McKnight Brain Institute, College of Medicine, University of Florida, Gainesville, FL 32610 USA; 5 Gene Therapy Center, University of North Carolina at Chapel Hill, Chapel Hill, NC 27599 USA; 6 Department of Chemistry and Biochemistry and Division of Biological Sciences, University of California-San Diego, La Jolla, CA 92093 USA

## Abstract

Single-particle cryogenic electron microscopy (cryo-EM) provides a powerful methodology for structural biologists, but the resolutions typically attained with experimentally determined structures have lagged behind microscope capabilities. Here, we exploit several technical advances to improve resolution, including per-particle contrast transfer function (CTF) refinement and correction for Ewald sphere curvature. The latter is demonstrated with several experimental samples and should become more standard as resolutions increase or at lower microscope accelerating voltages. The combined application of the described methods to micrographs recorded on a Titan Krios enables structure determination at ~1.86-Å resolution of an adeno-associated virus serotype 2 variant (AAV2), an important gene-delivery vehicle. The resulting structural details provide an improved model for understanding the biology of AAV that will guide future vector development for gene therapy.

## Abstract (web summary)

Single-particle cryo-EM is a powerful method for macromolecular structure determination. Here the authors demonstrate that Ewald sphere curvature correction, sub-Angstrom pixilation and per-particle CTF refinement can improve map quality and resolution and present the 1.86 Å cryo-EM structure of an adeno-associated virus serotype 2 variant.

## Introduction

Single-particle cryo-EM has become a powerful tool for macromolecular structure determination, owing largely to numerous technical advances over the past decade^1^. Whereas near-atomic resolution (~3–4 Å) can now be obtained routinely for well-behaved samples with limited conformational heterogeneity, achieving resolutions below ~2.5 Å remains challenging even for the most robust samples, and only two experimental cryo-EM structures have broken the nominal 2 Å barrier^2,3^. We sought to address a number of factors limiting the resolution of structure determination by single-particle cryo-EM. In these analyses, we studied a variant of adeno-associated virus (AAV) serotype 2 containing a single amino-acid substitution, L336C. The AAV2_L336C_ variant is of particular biological interest, as it is defective in genome packaging and is associated with reduced infectivity^4,5^.

AAVs are single-stranded DNA viruses that infect vertebrates^6^ and are thereby attractive vehicles for gene delivery^6,7^, with AAV2 being one of the most popular serotypes for such applications. The AAV viral capsid is formed by an icosahedral (*T* = 1)^6^ arrangement of 60 viral protein (VP) monomers, and has a molecular weight of ~3.9 MDa and an outer shell diameter of ~250 Å. The three related capsid proteins, VP1, VP2, and VP3, share a common core sequence and occur in a predicted 1:1:10 ratio^8^. AAV was particularly suited to our cryo-EM studies because: (1) it is relatively small for a virus and can be packed across cryo-EM grid holes in reasonably thin ice; (2) it can be stably assembled into homogeneous virus-like particles (VLPs) devoid of genomic material; and (3) it has icosahedral symmetry, which increases the number of asymmetric subunits in the dataset by 60-fold for each particle imaged.

Here we apply several technical advances in single-particle cryo-EM to the structure determination of the AAV2_L336C_ capsid variant, and we quantitatively evaluate the benefits of each, including an experimental demonstration of correcting for the curvature of the Ewald sphere. In addition to improving the structure of AAV_L336C_, correcting for the curvature of the Ewald sphere also improves the resolution for a reconstruction of the larger rotavirus double-layered particle. The results and described methods provide a feasible path toward more routinely obtaining structures at sub-2 Å resolution in single-particle cryo-EM.

## Results

### Data collection strategy

To image AAV2_L336C_ particles, we used a Titan Krios operating at 300 keV with a Gatan K2 Summit detector, without the use of technologies such as phase plates^9^, Cs correctors^10^, and energy filters^2^. For sample preparation, we used gold grids to reduce beam-induced movement^11^. For microscopy, we selected the smallest available C2 condenser aperture (70 μm on our Titan Krios) to maximize beam coherence, although this is not strictly necessary if the beam illumination is perfectly parallel. The spot size was selected to yield a beam diameter of ~2 μm to allow for complete illumination of the imaged hole, while ensuring that the imaged area is within range of parallel illumination^12^. During data acquisition, we used a relatively high magnification (37kx, corresponding to a pixel size of 0.788 Å) to boost the detective quantum efficiency (DQE) of the direct electron detector at a fixed spatial resolution^13,14^. Super-resolution mode reduced the final pixel size to 0.394 Å, which facilitates reaching close to or even beyond physical Nyquist in the reconstructed map^13,15^ while maintaining an otherwise identical field of view. During imaging, the microscope stage shift^16^ (rather than beam tilt-induced image shift) was used to center the specimen field of view so as to minimize beam tilt (and the concomitant effects of coma) within the final images, and a 60 s delay before the exposure was implemented to reduce grid movement due to thermal instability and hysteresis. A frame rate of 20 frames s^−1^ (50 ms frames) was used to correct for beam-induced motion^17^, although a running average of 3 sequential frames was subsequently determined to be an optimal setting for frame alignment. A dose of ~4 e^−^ pixel^−1^ s^−^^1^ on the K2 Summit detector was selected to reduce coincidence loss and maximize the camera detective quantum efficiency (DQE)^13^. With the above procedures, a coma-free microscope alignment allowed for observation of the 2.36, 2.04, and 1.44 Å line spacings in images of a gold-shadowed cross-grating replica calibration grid (Supplementary Fig. 1). This result indicated that the aligned microscope was capable of capturing high-resolution information within recorded images. While the above-mentioned settings may differ slightly between microscopes and facilities, to maximize data quality, employed procedures should result in high-resolution diffraction spots, as shown in Supplementary Fig. 1. Data collection throughput was aided by automation using Leginon^18^ across the 3.5-day-long session. We collected 1317 micrographs of AAV2_L336C_ particles, which after extensive pruning and classification (see Methods) provided a dataset of 30,515 particles for refinement (Supplementary Fig. 2 and Table 1).

**Table 1:** Cryo-EM data collection and modeling statistics

EM data collection/processing	AAV2_L336C_
**Microscope**	**FEI Titan Krios**
Voltage (kV)	300
Camera	Gatan K2 Summit
Nominal defocus range (μm)	0.6–2
Defocus mean ± std (μm)	1.1 ± 0.6
Exposure time (s)	3.5
Dose rate (e^−^ pixel^−1^ s^−1^)	4
Total dose (e^−^ Å^−2^)	22.5
Pixel size (Å)	0.788
Number of micrographs	1317
Number of particles (processed)	78,194
Number of particles (in final map)	30,515
Symmetry	I
Resolution limit during final refinement (Å)	1.90
Resolution (global) (Å)^a^	1.86
Local resolution range	1.78–1.92
Directional resolution range	1.86–1.86
Sphericity of 3DFSC	1
Map sharpening^b^	Spectral flattening between 8 and 1.8 Å, using pre-cut-off *b*-factor of −90 and post-cut-off *b*-factor of 0

Model statistics	AAV2_L336C_
Residue range	226–735
Map Cross Correlation	0.849
Root Mean Square Deviation RMSD [bonds] (Å)	0.01
Root Mean Square Deviation RMSD [angles] (Å)	0.93
All-atom clashscore	7.97
Ramachandran plot	
Favored (%)	97.2
Allowed (%)	2.8
Outliers	0
Rotamer outliers	0
C-β deviations	0
EM-Ringer score	8.23
Average *b*-factor	16.1

^a^Resolution assessment based on frequency-limited refinement using the 0.143 threshold for resolution analysis^61^

^b^Map sharpening based on *cis*TEM whitening and sharpening algorithm^23^

### Data processing strategy

**Fig. 1:**
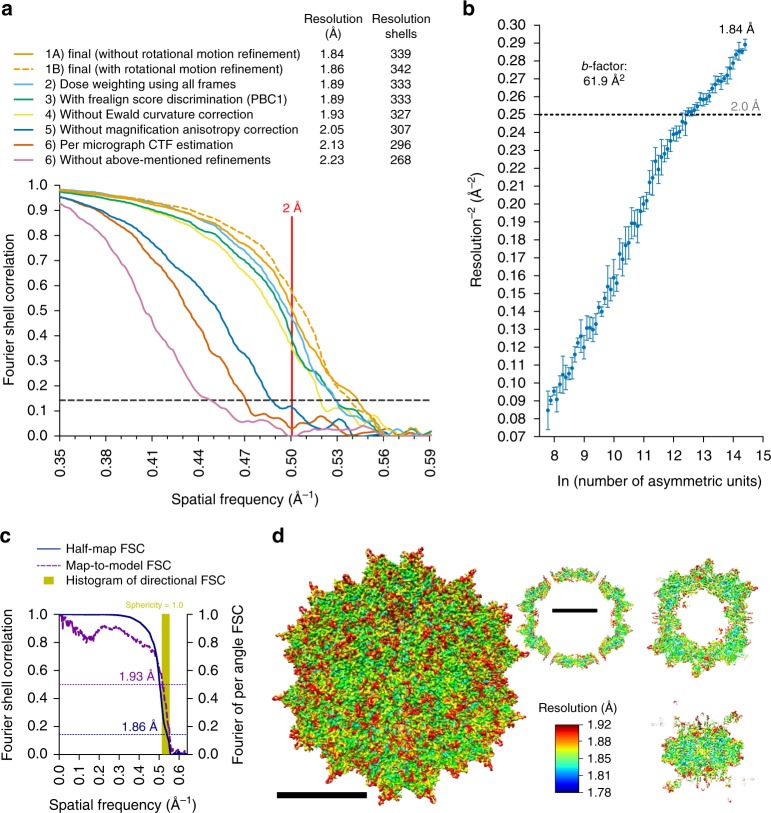
Procedures and implications for obtaining a sub-2 Å resolution reconstruction of AAV2_L336C_. **a** Fourier shell correlation (FSC) curves showing independent contributions of each operation to the final resolution. The resolution cut-off of 0.143 (gray dotted line) is indicated. Each FSC curve was generated by “turning off” one of the respective operations from the final refinement (1A). Correcting for rotational beam-induced movement (1B) improves the FSC at low spatial frequency. Discriminating particles based on their score (3) led to a lower resolution reconstruction (1.89 Å) as compared to equally weighting all particles (1.84 Å). Cumulative loss by “turning off” all the tested operations is indicated by (7). **b** ResLog^60^ plot of the dataset. Five replicates were performed for each data point with random particle distributions and the standard deviation is plotted. Linear regression was fitted and the *b*-factor^45^ indicated. **c** Fourier shell correlation (FSC) curves describing the half-map (blue solid line) and map-to-model (purple solid line) resolutions, as well as a histogram of directional resolutions sampled evenly over the 3DFSC^59^ (yellow), and corresponding sphericity value. The resolution cut-offs of 0.143 (blue dotted line) and 0.5 (purple dotted line) were used respectively. **d** 1.86 Å reconstruction of the AAV2 viral capsid using single-particle cryo-EM, colored by local resolution^56^. Slices through the reconstruction displayed in sub-panels start from the capsid center (left) move progressively towards the front (top right and bottom right). These show few deviations from the global resolution, with the best local resolution at 1.78 Å (primarily located inside the core region of the capsid shell). Both scales correspond to a distance of 100 Å

For data processing, multiple procedures resulted in improvements in resolution, as evidenced by changes across most frequency ranges within Fourier Shell Correlation (FSC^19^) curves, summarized in Fig. 1a. Improvements are described in spatial frequency shells, since at higher resolutions, gains are characterized by incrementally smaller increases in nominal resolution values. First, we removed particle images with the greatest angular uncertainty based on conventional scoring criteria in either Relion^20^ or Frealign^21^ and adjusted the weights for how different particles contribute to the reconstruction (see Methods). We then performed per-particle CTF estimation using GCTF^22^ and subsequently refined these values in *cis*TEM^23^, providing a cumulative gain of 46 resolution shells (~0.3 Å). Correcting for magnification anisotropy (estimated at ~1% on our Titan Krios)^24^ provided gains in resolution by 35 shells (0.21 Å). Notably, map resolution increased by 15 shells (0.09 Å) after correcting for the curvature of the Ewald sphere^25^, which has been predicted, but not previously demonstrated with experimental single-particle cryo-EM data (discussed further below). Additionally, per-frame reconstructions allowed us to determine which of the 70 frames contained the most information content. Reconstructions from individual frames (each receiving a dose of 0.32 e^−^ Å^−2^), provided maps with resolutions ranging between 2.1 and 3.4 Å (Supplementary Fig. 3a). Discarding the first 4 frames, which contained the largest beam-induced movement^17,26^ (Supplementary Fig. 3b), improved the map by 9 resolution shells (0.05 Å). Gold grids have been shown to reduce beam-induced motion as compared to carbon^11^, but the fact that we can still obtain quantifiable gains by eliminating the first frames suggests that further optimizing specimen supports^27^ should produce continued improvements to data quality. This is particularly true for advancing resolution beyond what is reported here, since the first frames contain the highest resolution structural information, which is quickly lost to radiation damage. Frames 5–19 could also be combined to produce a similar reconstruction to one composed from frames 5–70 (Fig. 1a and Supplementary Fig. 4). The remaining discrepancies, and the fact that frames 5–19 provided a slightly higher resolution map, is most likely due to minor differences in the measured compared to true curves describing global radiation damage to single-particle specimens. Finally, we found that correcting for the rotational particle movement through the course of the movie by refining the orientations of groups of five-frame averages improved low spatial frequency FSC values and the quality of the map, although the nominal value remained largely unchanged. Cumulatively, the above procedures resulted in a total gain of 71 resolution shells (~0.4 Å). As previously demonstrated^28^, the summation of individual gains is not equal to the cumulative improvement, as the effects are not necessarily additive.

### Ewald sphere curvature correction improves map resolution

The above results revealed that correcting for the curvature of the Ewald sphere is pertinent to experimental reconstructions in high-resolution single-particle cryo-EM analysis, warranting further investigation. Most 3D reconstruction algorithms assume that images correspond to projections of the 3D object, in accordance with the central slice projection theorem^29^. However, several aspects of cryo-EM data acquisition invalidate this approximation at resolutions approaching true atomic^30,31^. Most notably, imaged objects have a defined thickness along the optical axis of the microscope, and the top and bottom reside at different defoci with respect to the imaging plane, which results in an inherent focus gradient during imaging^32^. This intra-particle focus gradient alters the phases and amplitudes associated with each Fourier coefficient, and the effects become progressively more pronounced at higher resolutions, lower accelerating voltages, or for thicker specimens^33^. The way to correct for intra-particle focus gradients is to adjust the Fourier coefficients in a manner that maps them onto a characteristic surface that is described by the Ewald sphere^30^. Various schemes to estimate and correct for the curvature of the Ewald sphere have been developed^25,30–32,34,35^. Two^35,36^ experimental single-particle reconstructions have recently demonstrated improvements from taking Ewald sphere curvature into account, but this required manually breaking up the particles into subsets that are then independently reconstructed. A simpler approach, which corrects each particle image for the CTFs corresponding to either the right or left scattered beam, previously called the “simple-insertion” method for Ewald sphere curvature correction and implemented in Frealign9/*cis*TEM will insert the data for each particle image twice into correct Fourier coefficients related by Friedel symmetry^25^. This procedure, performed during the reconstruction, resulted in an increase of 15 resolution shells (~0.1 Å) within our final map (Fig. 1a).

**Fig. 2:**
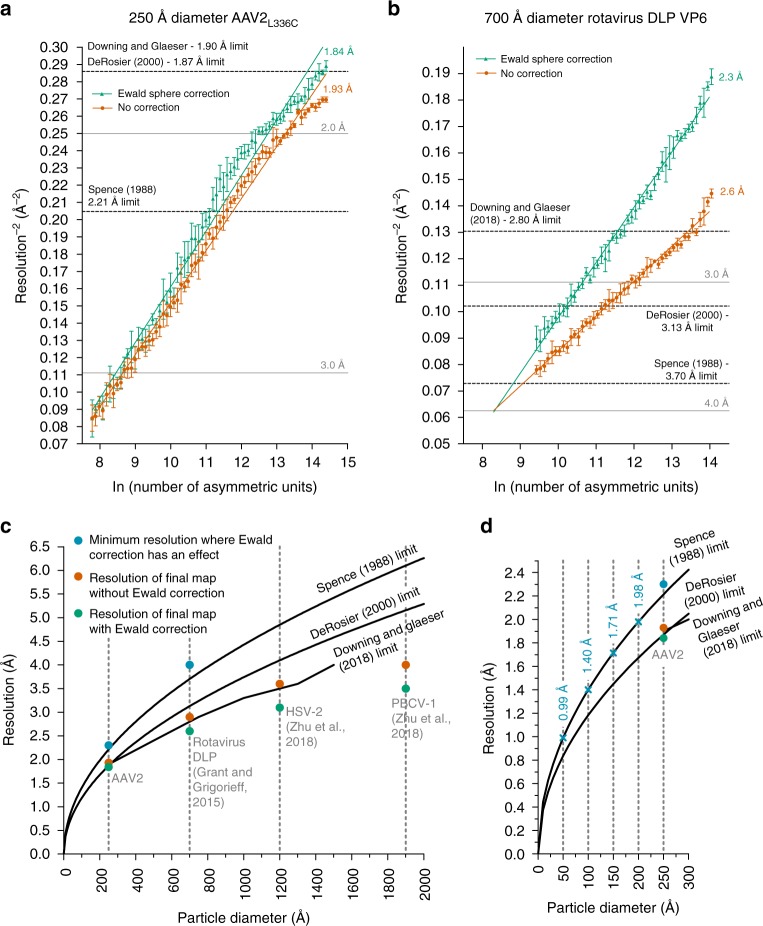
Ewald sphere curvature correction improves the resolution of AAV2_L336C_ and rotavirus VP6. ResLog^60^ plot of the **a** AAV2_L336C_ dataset and **b** rotavirus VP6 dataset from Grant and Grigorieff^37^, with (green) and without (red) Ewald sphere curvature correction in Frealign9. Five replicates were performed for each data point with random particle distributions, and the standard deviation is plotted. Linear regression is plotted for the first linear portion of each set of points. The resolution limits predicted by Spence^33,38^, DeRosier^30^, and Downing & Glaeser^32^ for a 250 Å and 700 Å macromolecule respectively are indicated. **c** The theoretical resolution limits caused by curvature of the Ewald sphere at 300 keV plotted against particle diameter. Reconstructions from this study and a previous study^35^ that have shown an improvement in final resolution with Ewald sphere curvature correction are plotted (orange with and green without Ewald sphere curvature correction). The minimum resolutions at which noticeable improvements due to Ewald sphere curvature correction occur are also plotted in blue for AAV2_L336C_ and rotavirus VP6, and follow closely with the Spence limit. **d** The theoretical resolution limits caused by the curvature of the Ewald sphere for particles smaller than 300 Å in diameter at 300 keV. Points on the Spence limit corresponding to 50, 100, 150, and 200 Å particle diameters are indicated and labeled in blue. Rest of the coloring scheme is the same as in **c**

We then evaluated the effect of Ewald sphere curvature correction at lower resolution by reducing the number of particles in the reconstruction. Randomly selected subsets of the data containing an approximately equal defocus range were used to perform reconstructions with incrementally smaller numbers of particles. As few as ~60 particles (3600 asymmetric units) were sufficient to produce a ~3.5 Å map, whereas ~120 particles (7200 asymmetric units), and all larger subsets, were sufficient for <3 Å reconstructions (Supplementary Fig. 5). These maps could be used to evaluate Ewald sphere curvature effects as a function of resolution for the ~250 Å diameter particle. Noticeable gains appeared at ~2.4–2.3 Å, and a final improvement of 15 shells (~0.1 Å) for the best reconstruction (Fig. 2a). The gains follow an increasing trend at higher resolution, as the effects of Ewald curvature become more pronounced at higher electron scattering angles^30^. Furthermore, the correct handedness of a reconstruction can be explicitly determined when accounting for the effects of the Ewald sphere^25^. Specifically, the handedness of the reconstruction defines how the Fourier coefficients are substituted in the reconstruction and whether an inversion operation must be applied to the Fourier coefficients (Supplementary Fig. 6).

The modest resolution gains of ~0.1 Å for AAV2_L336C_ are likely due its small diameter of ~250 Å. As a comparison, we used Grant et al.’s^37^ dataset of a larger rotavirus double-layered particle (DLP), which is 700 Å in diameter, and performed a series of Ewald sphere curvature-corrected reconstructions using a decreasing number of particles (Fig. 2b). The resolution gains were more pronounced—noticeable gains appear above 4 Å resolution with experimental data that was icosahedrally (but not *T* = 13) averaged. Using the full dataset, we observed a resolution gain of ~0.3 Å with icosahedral averaging (2.9 versus 2.6 Å), as well as with icosahedral and *T* = 13 averaging (2.6 versus 2.3 Å). This trend continues for even larger particles reported by Zhu et al.^35,36^. with Ewald sphere curvature correction (implemented by breaking up particles into chunks that could be separately reconstructed), the 1200 Å wide HSV-2 had a resolution gain from 3.6 to 3.1 Å, whereas the 1900 Å diameter PBCV-1 improved from 4 to 3.5 Å. Different estimates have been developed to approximate the resolution limits in the absence of Ewald sphere curvature correction, including by Spence^33,38^, DeRosier^30^, and Downing & Glaeser^32^, which effectively differ in the amount of phase errors that can be tolerated within a reconstructed object. Correcting for Ewald sphere curvature appears to begin producing experimental benefits at a point when the phase shift of the electron wavefront from the top to the bottom of a particle (with a defined diameter) is ~180° (*π*), which is consistent with the original Spence estimate (Fig. 2c, blue circles). In the absence of Ewald sphere curvature correction (Fig. 2c, orange circles), errors seem to be reasonably well tolerated for icosahedral particles, possibly due to the large amount of symmetry and thus the amount of “correct” Fourier coefficients in every view. However, correcting for Ewald curvature further improves the quality and resolution of all reported reconstructions (Fig. 2c, green circles). The existing curves could possibly be used to approximate the resolution at which a particle can benefit from Ewald sphere curvature correction, although further studies, for example with asymmetric particles and at different microscope voltages, would have to be performed to determine where the effects would have the most significant impacts. It is clear that such effects will become increasingly more important for particles smaller than 200 Å in diameter at increasingly higher resolutions (Fig. 2d), as well as for lower microscope accelerating voltages, such as 120 or 200 keV^34,39^.

### Contribution of particles from different defoci

Particles further from focus are thought to result in a lower resolution reconstruction due to dampening of the envelope function^40^, while particles closer to focus might not have enough contrast for proper alignment. The AAV_L336C_ dataset was collected with a defocus range of 0.4–2.5 μm (centered at 1.1 ± 0.6 μm). Interestingly, only slight differences were observed when comparing the FSCs of reconstructions from particles at different estimated defoci, although it was necessary to use a sufficiently large box to avoid aliasing effects (Supplementary Figure 7). These results indicate that, at least with AAV particles and given the nominal defocus range on a 300 keV microscope equipped with a field emission gun, data collected further from focus may not be as compromised by illumination-coherence effects of the microscope envelope, provided that a sufficiently large box is used for windowing the particles^41^ (see Methods) to avoid truncating spatial information that has been delocalized due to defocusing.

### High-resolution details within the AAV2_L336C_ cryo-EM reconstruction

**Fig. 3:**
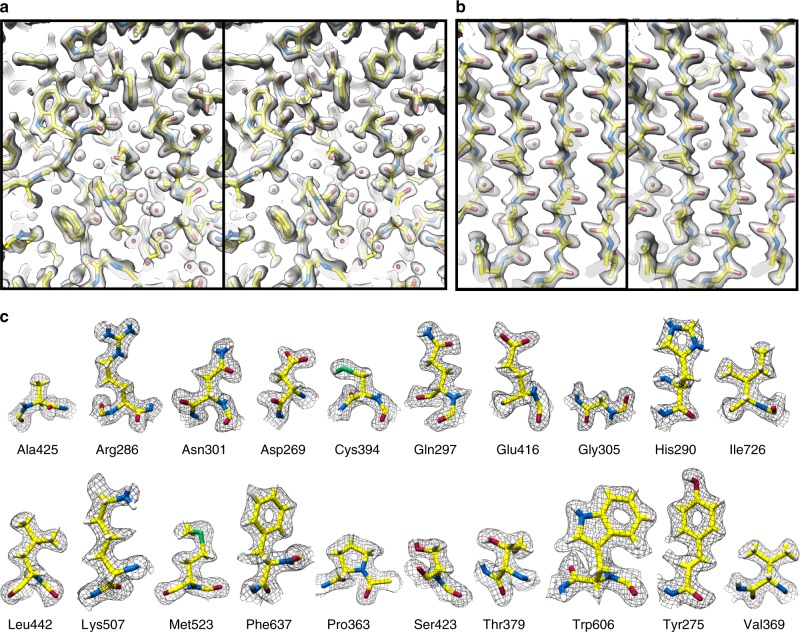
High-resolution information within the 1.86 Å reconstruction of AAV2_L336C_. **a** Stereo view of a slice through the map and model containing both amino-acid residues and water molecules. **b** Stereo view of a slice through a beta sheet. **c** Map densities for each of the 20 types of amino-acid residues. The amino-acid residues are shown as stick representation and colored according to atom type: *C* = yellow, *O* = red, *N* = blue, *S* = green inside either a translucent solid density (**a**, **b**) or black mesh density map (**c**). *H* = white atoms are displayed for **c**. The scale bars correspond to 10 Å

The final map of AAV2_L336C_ had a global resolution of 1.86 Å, with a largely homogeneous local resolution distribution within the core of the capsid shell that drops to ≥1.92 Å at the solvent-exposed surfaces (Fig. 1c–d and Table 1). Using this map, an atomic model was derived for the common region of the VP monomer, residues 226 to 735 (VP1 numbering), which was symmetry expanded by icosahedral matrix multiplication to produce the full 60-mer viral capsid. As in previously reported AAV structures, the VP1u, VP1/2 common sequence, and the N terminus of common VP3 are disordered. The final model corresponded closely to the map, with good statistics (Table 1), including a high EM-Ringer^42^ score of 8.23 and a correlation coefficient following model refinement in Phenix of 0.849^43^. The EM-Ringer score reflects accuracy of fit between model and map based on side-chain rotameric positions. At this resolution, the map is of sufficient quality to see numerous features with unprecedented detail (Fig. 3 and Supplemental Movie 1) including: (1) the backbone tracing with well-defined carbonyls; (2) explicit structure to most side-chains, rotamers, holes in aromatic residues, as well as prolines and associated puckers; (3) ordered solvent throughout the structure, including primary and secondary hydration shells; (4) the distinct appearance of density for individual oxygens of carboxylate groups, which occasionally begin to show traces of H-bonding geometry, and (5) appearance of density that corresponds to the first indications of hydrogen atoms (Supplementary Figure 8).

### Comparison of structures of AAV2_L336C_ with AAV2_WT_

**Fig. 4:**
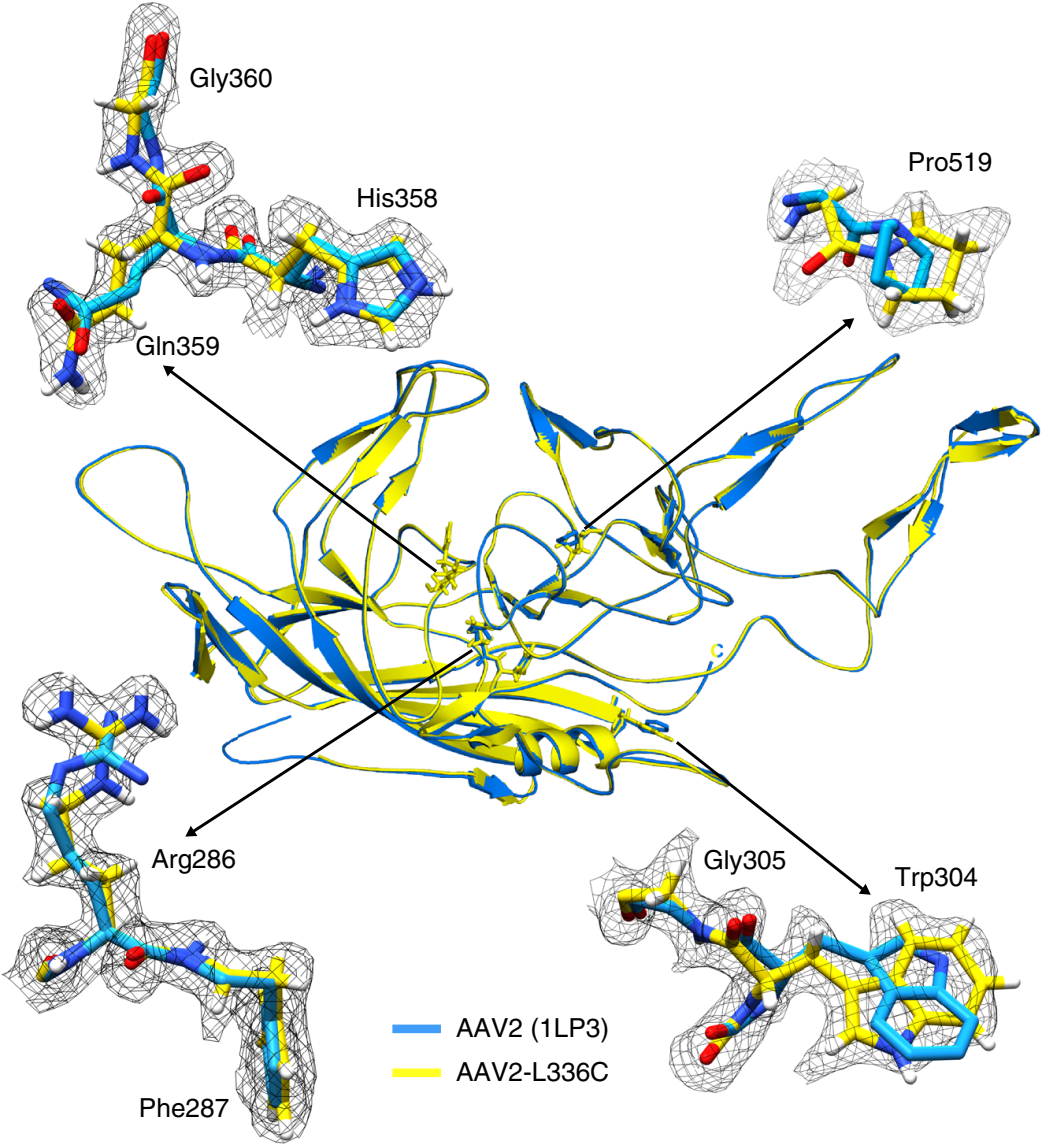
High-resolution map aids in placement of correct rotamers for AAV2_L336C_. At 1.86 Å (map in black mesh), uncertainty in rotamer and backbone conformation in a previous model of AAV2 (1LP3, in cyan) determined by X-ray crystallography to 3 Å resolution can now be accurately modeled (yellow)

**Fig. 5:**
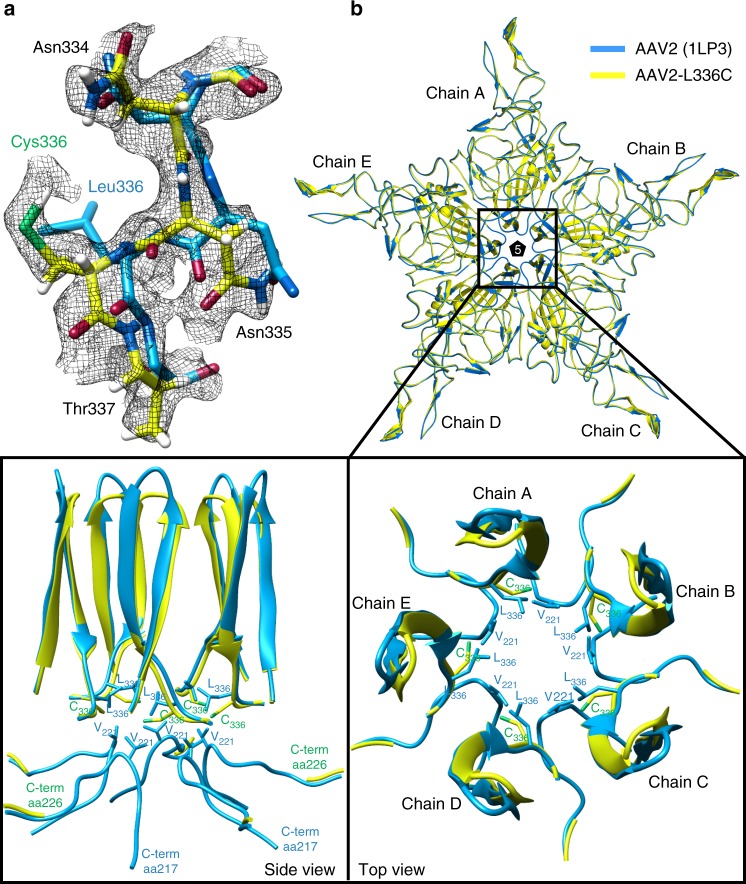
Comparison of AAV2_WT_ and AAV2_L336C_. **a** The density map with the modeled residues for the DE-loop for AAV2_WT_ and AAV2_L336C_. The wild-type L336 and substituted C336 residues are shown. The AAV2_WT_ has been the highest resolution density map for AAV2 published to date, at 3 Å. The amino-acid residues are shown as stick representation and colored according to atom type: *C* = yellow, *O* = red, *N* = blue, *H* = white, *S* = green inside a black mesh density map. **b** Superposed pentamer models of AAV2_WT_ (blue) and AAV2_L336C_ (yellow). Lower panel show close-up views of the fivefold region as side view and top view perspectives. Side-chain atoms for individual residues of interest are shown

The density for C336 is clearly ordered in the AAV2_L336C_ map, and the model is considerably improved compared with the prior structure of AAV2_WT_ (Fig. 4)^44^. There is a 1.4 Å shift of the main-chain of C336 and neighboring residues, compared to AAV2_WT_ (Fig. 5a). This results in a 0.8 Å widening at the base of the fivefold channel formed by five symmetry-related DE loops (the loop between the βD and βE strands). In addition, the AAV2_WT_ structure is ordered from residue 217 to 735, with the additional N-terminal residues compared to AAV2_L336C_ (residues 226 to 735) occupying the base of the interior opening of the fivefold channel (Fig. 5b). The AAV2_L336C_ variant displays a 2–3-fold defect in genome packaging compared to AAV2_WT_ and lacks PLA2 activity resulting in a defect in infectivity^4,5^. This defect was proposed to be due to the inability to expose the PLA2 and potential structural differences to AAV2_WT_. The annotated differences in AAV2_L336C_ support these possibilities. An altered location of the PLA2 domain due to the N-terminal disorder would abrogate its externalization via the 5-fold pore and thus its function.

## Discussion

Efforts to improve the AAV gene delivery system have focused on structure–function analysis of the viral life cycle and of engineered capsids to improve therapeutic efficacy. The 1.86-Å resolution structure of AAV2_L336C_ represents the most accurately interpreted AAV capsid model thus far (Fig. 4). This particular variant also exhibits specific structural changes that are clearly captured in the density map, and these changes may be associated with infectivity defects (Fig. 5). High-resolution structural information can aid the annotation of: (1) water networks required to stabilize the capsid structure assembly and involved in its function; (2) the protonation states of acidic and histidine residues important for interactions in the endo/lysosomal pathway; (3) capsid interactions with the transcription machinery and during capsid assembly; and (4) precise receptor and antibody interactions. Details from such analyses can guide the engineering of AAVs at specific residues to eliminate interactions, such as those with pre-existing host immune system molecules, or improve function, such as specific tissue targeting. Notably, the number of known sites of interaction between the AAV ligand and host receptor/antibody far exceeds the number of experimentally derived AAV models. For this reason, the fact that high-resolution structures of AAV variants can be derived with as few as ~100 particles (Supplementary Fig. 5) is noteworthy and will accelerate the compilation of a comprehensive structural understanding of AAV:host interactions^6^.

The workflow described herein provides a feasible route toward true atomic resolution in cryo-EM single-particle analysis. While we used a well-behaved sample for this work, the modest amount of time (3.5 days collection) and equipment (a Titan Krios and K2 Summit camera) used for this reconstruction would make our strategy generally applicable, even though the relative gains will differ by specimen (Supplementary Fig. 9). Correcting for the curvature of the Ewald sphere should be incorporated into reconstruction algorithms. Finally, our sub-2 Å resolution reconstruction of AAV2_L336C_ also provides new insights into the AAV life cycle and biology that will be invaluable for improving the effectiveness of AAV as a delivery vehicle in gene therapy applications.

## Methods

### Statistics

For calculations of Fourier shell correlations (FSC), the FSC cut-off criterion of 0.143^45^ was used.

### Production and purification of AAV2_L336C_ virus-like particles

The AAV2_L336C_ substitution was created within the AAV2 *cap* gene encoding all three viral proteins, VP1, VP2, and VP3, as previously described^5^. A recombinant baculovirus, encoding the AAV2 *cap* gene, with the L336C substitution, was created using the Bac-to-Bac system (Thermo Fisher). A plaque purified and titered baculovirus stock was used to infect *Sf*9 insect cells, at a multiplicity of infectivity of 5 to generate virus-like particles (VLPs). The harvested pellet (from lysed cells and polyethylene glycol precipitated supernatant) was freeze/thawed three times with Benzonase (EMD Millipore Cat#712053) treatment. After the third thaw, the resulting clarified supernatant was purified using a step iodixanol gradient followed by anion exchange^46^ and then dialyzed into 50 mM HEPES, pH 7.4 with 2 mM MgCl_2_ and 150 mM NaCl. The sample concentration was determined by optical density assuming an extinction coefficient of 1.7 mg mL^−1^ cm^−1^) for AAV2 VLPs. The VLP purity and integrity were confirmed by sodium dodecyl sulfate polyacrylamide gel electrophoresis and negative stain EM on an FEI Spirit TEM, respectively.

### Single-particle CryoEM vitrification

Double blotting was used to increase particle concentration^47^. A 2.5 μL aliquot of AAV2_L336C_ sample at 2.5 mg mL^−1^ was added to a plasma-cleaned (Gatan Solarus) 1.2 µm hole, 1.3 µm spacing holey gold grid (Quantifoil UltrAuFoil) and blotted away using filter paper after 20 s wait time. Another 2.5 μL of the same sample was then re-applied to the grid and blotted after 20 s wait time and then vitrified in liquid ethane using a manual plunger. All operations were performed in a 4 °C cold room at >80% humidity to minimize evaporation and sample degradation.

### Data acquisition

Images were recorded on a Titan Krios electron microscope (FEI/Thermo Fisher) equipped with a K2 summit direct detector (Gatan) at 0.394 Å per pixel in super-resolution counting mode (0.788 Å for the physical pixel size) using the Leginon software package^18^. Data collection was performed using a dose of ~22.5 e^−^ Å^−2^ across 70 frames (50 m frame^−1^) at a dose rate of ~4 e^−^ pix^−1^ s, using a set nominal defocus range of −0.6 to −2 μm. On our microscope, the 100 μm objective aperture allows for transmission of information up to ~1.4 Å, but could not be aligned to produce a coma-free diffractogram. In contrast, the 70 μm aperture would truncate information at the ~2 Å limit. For this reason, the objective aperture was removed to prevent physical truncation of the most widely scattering electrons—and thus the highest resolution information. A total of 1317 micrographs was recorded over a single 3.5-day collection.

### Data processing

Movie frames were aligned using MotionCor2^48^ with 5 by 5 patches, a grouping of 3 and B-factor of 100, and Fourier space binning of 2 (resulting in a pixel size of 0.788 Å pixel^−1^) through the Appion software package^49^. Micrograph CTF estimation was performed using both CTFFind4^50^ for whole micrographs and GCTF^22^ for individual particles within the Appion software package. A subset of eight micrographs was first used for particle picking using Gautomatch (Kai Zhang, unpublished, https://www.mrc-lmb.cam.ac.uk/kzhang/Gautomatch/), and particles were extracted and analyzed by 2D classification in Relion 2.1^20^. 2D class averages that showed clear structural details were used as templates for template-based picking using Gautomatch on all 1317 micrographs. A total of 78,194 particles were then extracted using a box size of 800 pixels and subjected to two initial rounds of 2D classification (binned by 4) to identify and discard false positives such as ice and other obvious contaminants. The selected box size avoids aliasing spatial information up to 1.8 Å that was delocalized due to defocusing up to −3.2 μm^41^. Following 2D classification, 36,620 particles were re-extracted with the re-centering option in Relion.

3D refinements were performed first using Relion and finishing in *cis*TEM^23^, with the initial model generated by CryoSPARC^51^. Icosahedral symmetry was imposed during all 3D refinement steps, based on prior knowledge^6^ of AAV2 structure. All conversions between Relion, CryoSPARC, and *cis*TEM were performed using Daniel Asarnow’s pyem script (unpublished, https://github.com/asarnow/pyem). An initial 3D refinement using 7 rounds of auto-refinement and 2 rounds of local refinement was performed with binning of 2. Particles were discarded based on analysis of the “score” values in *cis*TEM leading to the removal of a distinct subset of particles with low scores (below 6 in this dataset). This resulted in 30,515 particles that were re-extracted, unbinned, and used for all subsequent operations. Per-particle CTF refinements were performed within *cis*TEM. All final refinements used a ring-shaped mask with an inner diameter of 75 Å and an outer diameter of 150 Å to specifically include only the capsid density and exclude remaining solvent. For this dataset, and after applying the stack-filtering procedures described above, 3D classifications did not produce any noticeable further gains.

Plotting the defocusV against defocusU values^52^ showed a systematic scaling of the difference between these two values as a function of their magnitude. Using the mag_distortion_estimate software^24^ and micrographs collected from a gold-coated cross-grating replica grid (Supplementary Fig. 1), a magnification anisotropy of 1.10% was calculated. The appropriate correction for magnification anisotropy was applied during frame alignment (see above). The particle stack that was re-extracted from magnification anisotropy-corrected frame sums reached a resolution of 1.97 Å after derivation of an ab initio model in CryoSparc and refinement in *cis*TEM. *cis*TEM and Frealign9 also allow for adjusting the weights for how the particles are inserted into the reconstruction. Low-scoring particles were down-weighted compared to high-scoring molecules. Likewise, it is possible to weight the particles by defocus. In this dataset, we did not find any correlation between the score value and the defocus of the particle (Supplementary Figure 7). For this reason, no weighting was performed, and we accordingly found that the resolution of the map was improved without applying any weighting to the particles (Fig. 1a).

The aligned movie frame stack was also split into individual frames and using the best Euler angles and shifts from above, reconstructions were computed. Frames 5–19, each of which independently exceeded a resolution of 2.24 Å, were summed and used for subsequent manual refinement (including CTF refinement) within *cis*TEM to obtain a reconstruction at 1.93 Å. The final reconstruction at 1.84 Å was computed after correcting for the curvature of the Ewald sphere. Rotational motion correction was performed in *cis*TEM by splitting each particle sum into groups of 5 frames (frames 5–9, 10–14, and 15–19), and refining each group-of-5 as if it were a single particle. Particle-frame-averages with score lower than 3 were removed, resulting in a final stack of 87,781 “particles” that refined to 1.86 Å resolution. Although the nominal resolution at 0.143 cut-off was worse than without rotational frame alignment, inspection of the FSC curves and visual comparison of the two maps suggested that this procedure provided minor benefits. Notably, information at lower spatial frequencies was slightly improved within the reconstruction following rotational frame alignment. This final map was then up-sampled by a factor of 2 (clipped to 360 box size and up-sampled to 720) in order to better visualize high-resolution features and fully represent all Fourier components within the real-space map.

To generate maps of the opposite handedness, Euler angles of the particles were changed from (phi, theta, psi) to (-phi, 180-theta, psi). Ewald sphere curvature-corrected reconstructions of same and opposite handedness were performed by setting IEWALD to either 1 or −1, respectively, in Frealign9.

For generation of the ResLog plots for both AAV2_L336C_ and VP6 rotavirus, five replicates were performed for each data point (selected evenly on a logarithmic scale) with random particle distributions. For the VP6 rotavirus, one round of *cis*TEM CTF refinement was performed on the stack from Grant and Grigorieff^37^ before generation of the ResLog plot, and the 13-fold averaging was performed as per the original study.

### Model refinement

For model refinement of the AAV2_L336C_ variant, the deposited structure of AAV2 (PDB-ID: 1LP3 [10.2210/pdb1LP3/pdb]) was used as a starting template. A 60-mer capsid model downloaded from VIPERdb^53^ (http://viperdb.scripps.edu) was docked into the map using the “Fit-in-map” function in the Chimera^54^ program. To optimize the correlation coefficient (CC) between the model and map the voxel (pixel) size of the map was adjusted. From the fitted 60-mer, a monomer was extracted for the model building. For model building and real-space refinement in the Coot^55^ program, the map was converted from the Purdue Image Format (PIF) to the XPlor format using e2proc3D.py subroutine in the EMAN2^56^ application and finally to the CCP4 format using MAPMAN^57^. In Coot^55^, L336 in AAV2_WT_ was substituted to a cysteine and the side-chain and main-chain atoms, including those of neighboring residues, adjusted to better fit the experimental density map using the real-space-refinement subroutine. After the manual refinement of the monomer was completed, a 60-mer was regenerated in VIPERdb by *T* = 1 icosahedral matrix multiplication. The model was further real-space refined against the cryo-EM map with Phenix^43^ utilizing the default settings, which includes strict NCS constraints, rotamer, and Ramachandran restraints in real space, and B-factor refinement subroutines of the program. The CC and refinement statistics, including root mean square deviations (RMSD), bond lengths and angles were analyzed by Phenix. Model adjustment and refinement were performed iteratively in Coot and Phenix, and the statistics were examined using Molprobity^58^ until no further improvements were observed. The final map and model were then validated using (1) EMRinger^42^ to compare map to model, (2) SPARX^56^ to calculate map local resolution and (3) 3DFSC program suite^59^ to calculate degree of directional resolution anisotropy through the 3DFSC.

## Electronic supplementary material

Supplementary Information

Editorial Note

Description of Additional Supplementary Files

Supplementary Movie 1

## Data Availability

All raw movie frames, micrographs, the particle stack, and relevant metadata files for the AAV2_L336C_ dataset are deposited in the EMPIAR database as EMPIAR-10202. The electron-density map of AAV2_L336C_ is deposited into the electron microscopy databank as EMD-9012. The model has been deposited into the PDB as PDB-6E9D. Other data are available from the corresponding authors upon request.
