# Supplementary material for: Sub-2 Å Ewald curvature corrected structure of an AAV2 capsid variant
Source: Nat Commun. 2018 Sep 7;9:3628. doi: 10.1038/s41467-018-06076-6 (PMC6128836; doi:10.1038/s41467-018-06076-6)
Supplement: Supplementary file 1 — Supplementary Information [file 41467_2018_6076_MOESM1_ESM.pdf]

Supplementary Information for:

Sub-2 Å Ewald Curvature Corrected Structure of an AAV2 Capsid Variant

Correspondence and requests for materials should be addressed to M. A.–M. (email: mckenna@ufl.edu) or to D. L. (email: dlyumkis@salk.edu)

Supplementary Figures

Supplementary Figure 1 | High-resolution information is present within micrographs. Zemlin tableau recorded under cryogenic conditions showing power spectra of images of a gold-coated carbon cross-grating replica grid, acquired with a 5 mrad beam tilt in the respective direction, and subsequent to coma-free microscope alignment. Gold diffraction spots at 2.355 Å (averaging to a nearly concentric ring), 2.039 Å, and 1.442 Å are evident within the series of power spectra. Arrows point to faint diffraction spots visible at 1.442 Å.

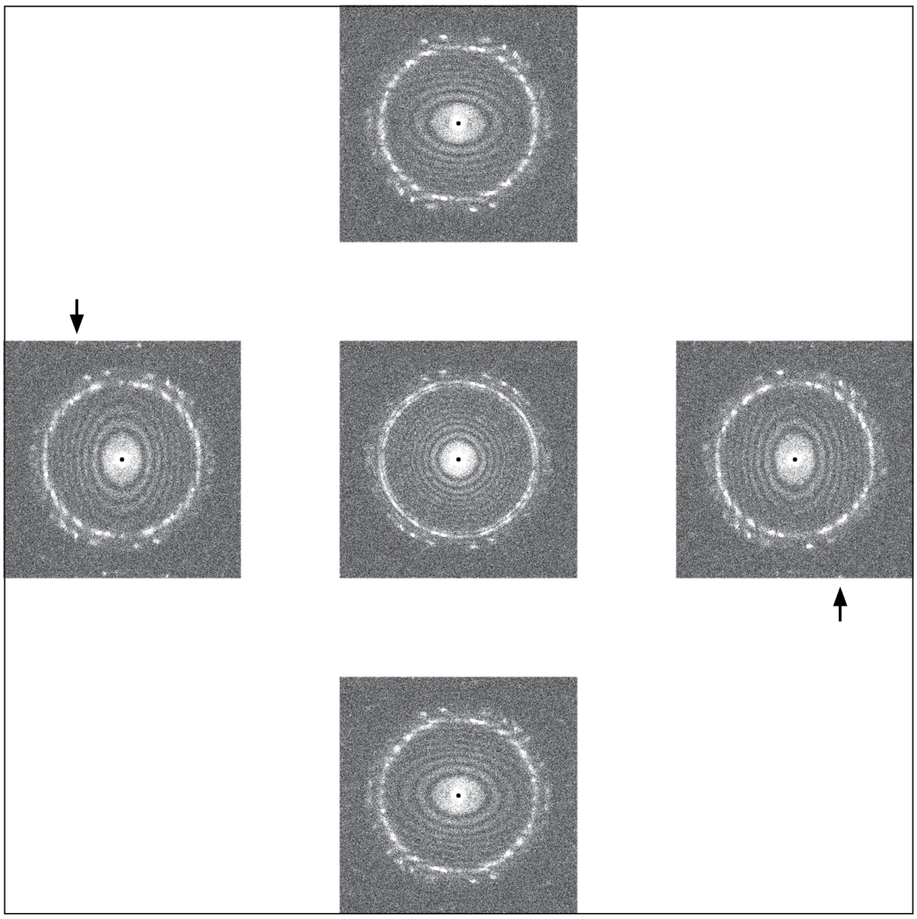

Supplementary Figure 2 | Raw cryo-EM data. (**a**) A cryo-EM micrograph of the $AAV2_{L336C}$ imaged on a Titan Krios with a Gatan K2 summit direct detector at -1.19 µm defocus, estimated using CTFFind4. The length of the scale bar is 20 nm. (**b**) CTF estimation profile from Appion[1]. (**c**) 2D class averages of $AAV2_{L336C}$ aligned using Relion. The length of the scale bar is 35 nm.

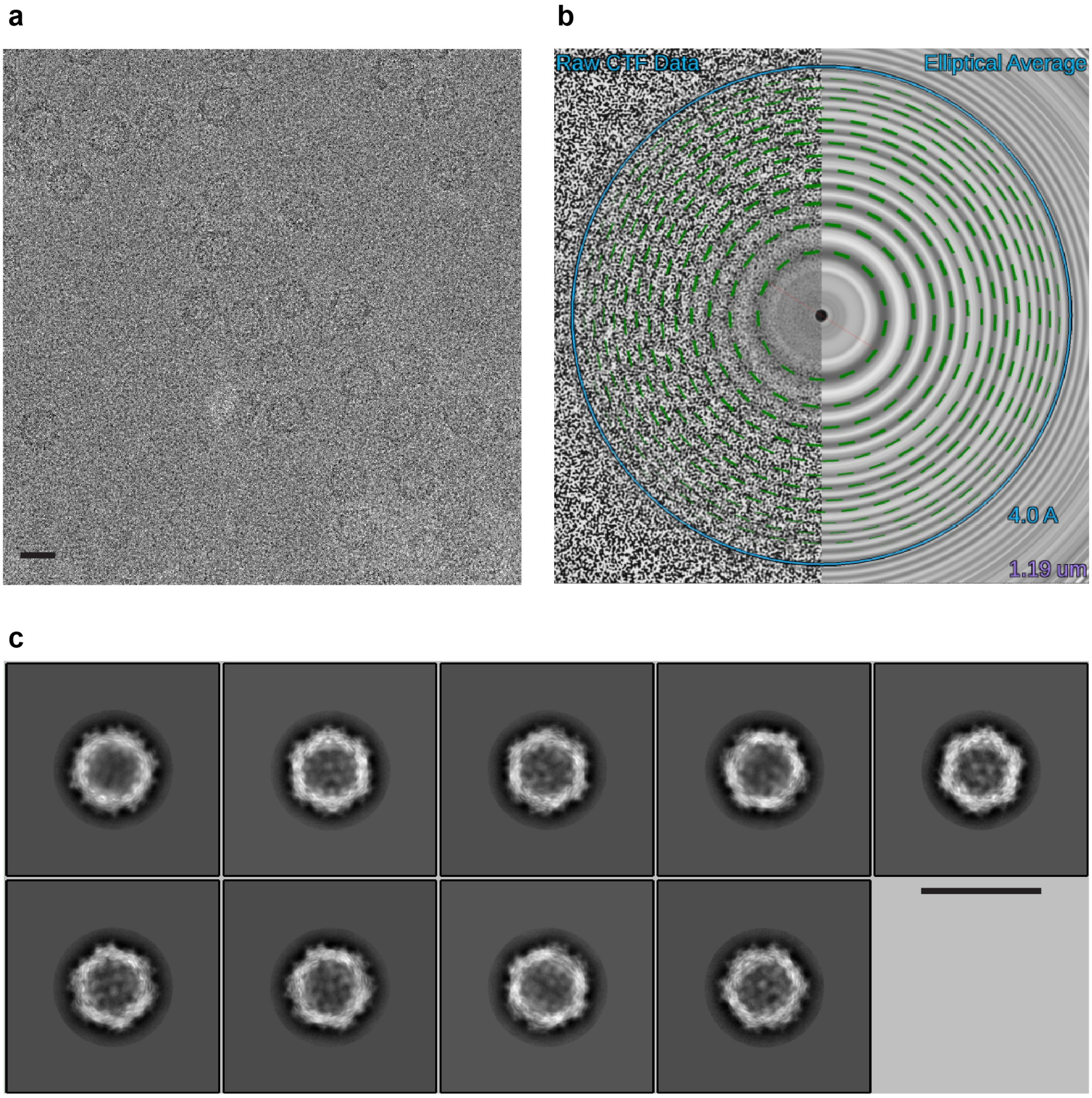

Supplementary Figure 3 | Resolution of individual frame reconstructions. (**a**) Using the best Euler angles and shifts, reconstructions were computed separately for each of the 70 frames. The resulting resolution shows two trends: the first 4 frames (3.17-2.43 Å) suffered from the initial effects of beam-induced motion; after frame 22, the resolution gradually worsens owing to the cumulative effects of radiation damage. (**b**) Frame-to-frame shifts in Ångstroms for all 70 frames are shown in blue. Frame-to-frame shifts were calculated using MotionCor2 global frame alignment mode.

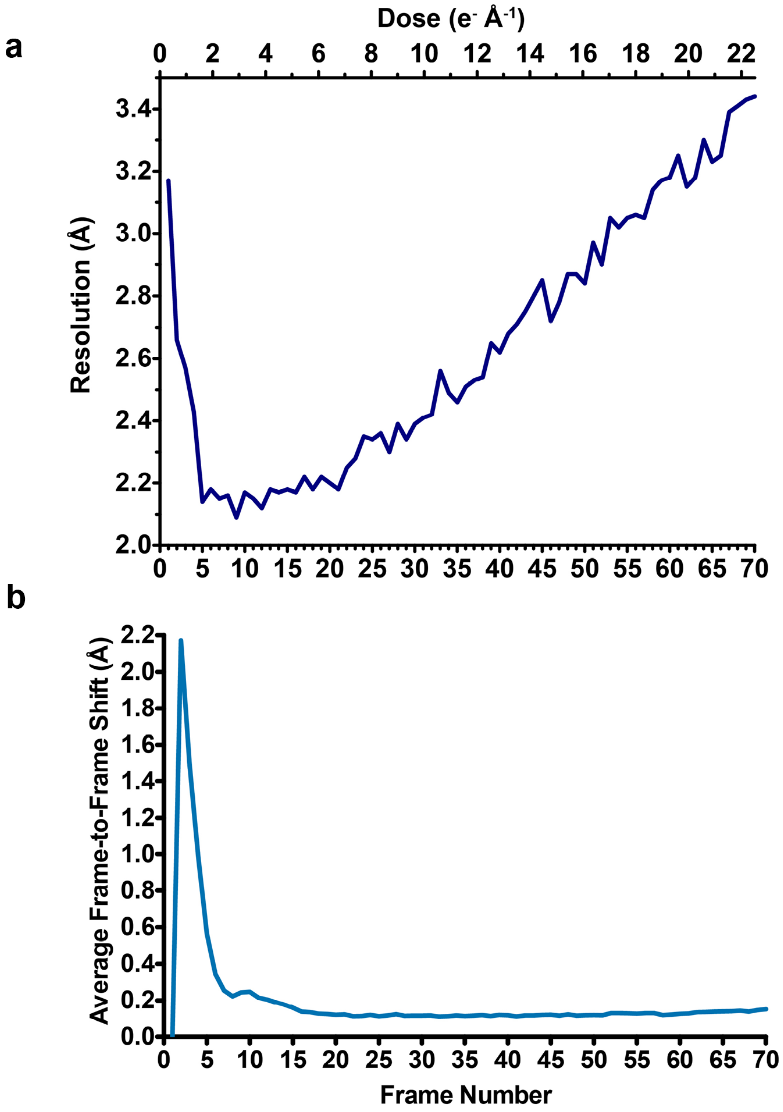

Supplementary Figure 4 | Effect of removal of first frames. Removing the first 4 frames that exhibit the most beam-induced movement, and consequently the lowest resolution (see Supplementary Fig. 3), either from the subset of frames 5-19 (2) or from the cumulative, dose-weighted sum containing frames 5-70 (3) produces measurable resolution gains.

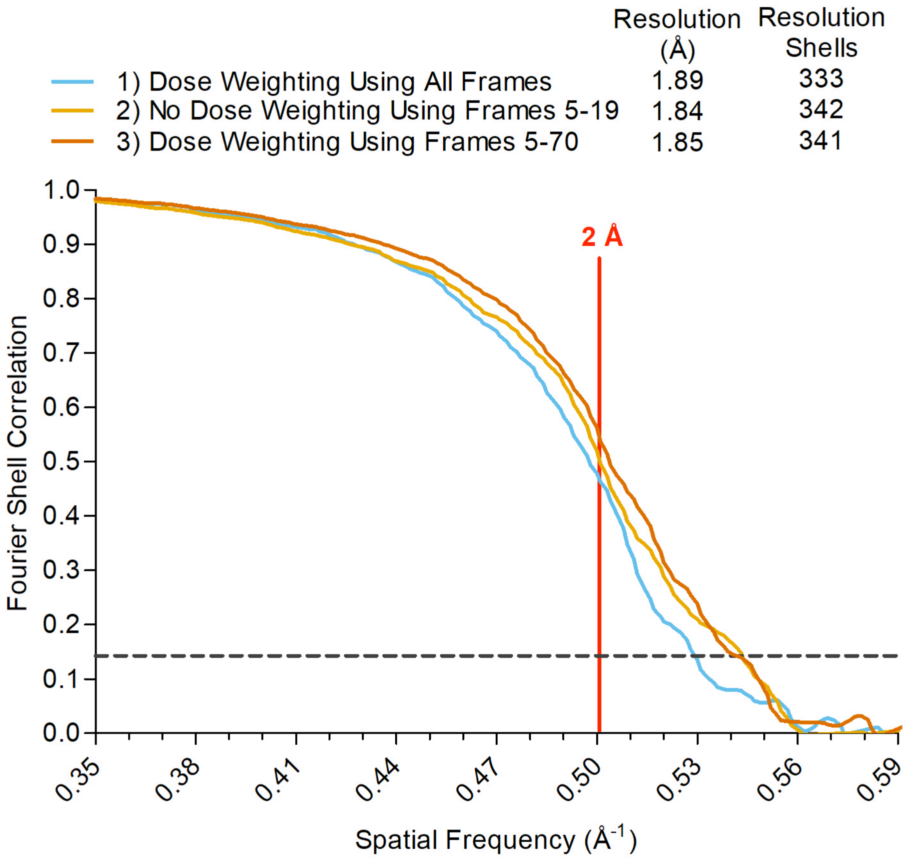

Supplementary Figure 5 | Improvement in resolution with increasing particle number. Reconstructions were performed with subsets of the particles, before rotational motion correction. (**a**) FSC curves, (**b**) a plot of nominal resolution value (expressed in Å^{-2}) as a function of the number of asymmetric units (ASUs), and (**c**) views of the density maps showing structural features for each corresponding reconstruction. Water molecules and holes in aromatic residues become obvious at beyond 2.10 Å.

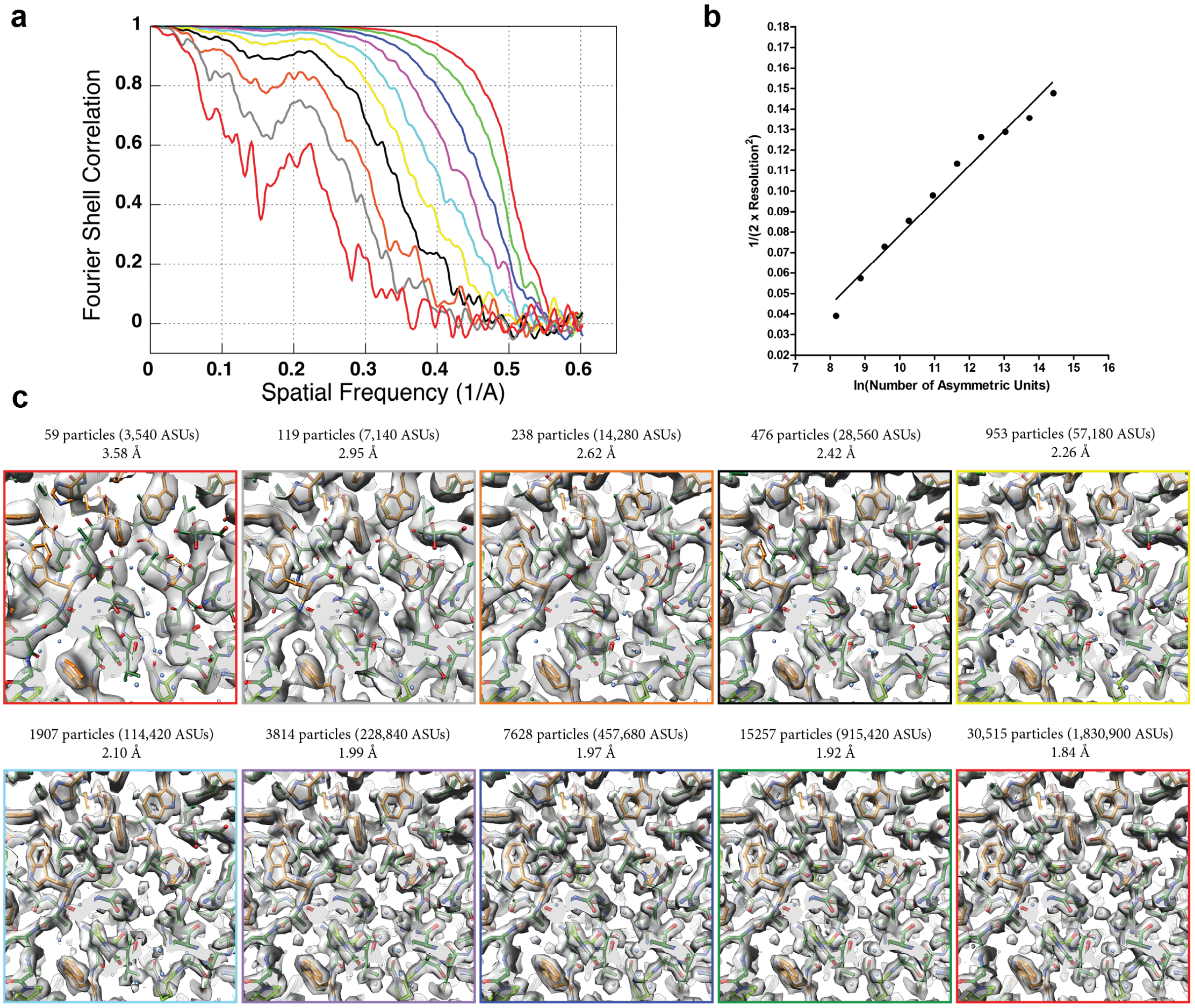

Supplementary Figure 6 | Accounting for Ewald sphere curvature improves cryo-EM resolutions and allows for determination of map handedness. A map reconstructed without correcting for the curvature of the Ewald sphere and using orientation parameters accounting for (1) the correct or (4) opposite map handedness have identical resolutions. Accounting for the curvature of the Ewald sphere during the reconstruction for the correct-handed map improves resolution (2), whereas the same operation performed on a reconstruction of the opposite-handed map decreases the resolution (5). Analogously, an inversion of the Fourier coefficients during Ewald curvature correction, if applied to the correct-handed map, decreases resolution (3), whereas this same operation performed during the reconstruction of the opposite-handed map effectively restores all high-resolution components (6). These sets of operations allow for automated determination of handedness in high resolution cryo-EM maps in the absence of auxiliary data, as previously predicted[2,3].

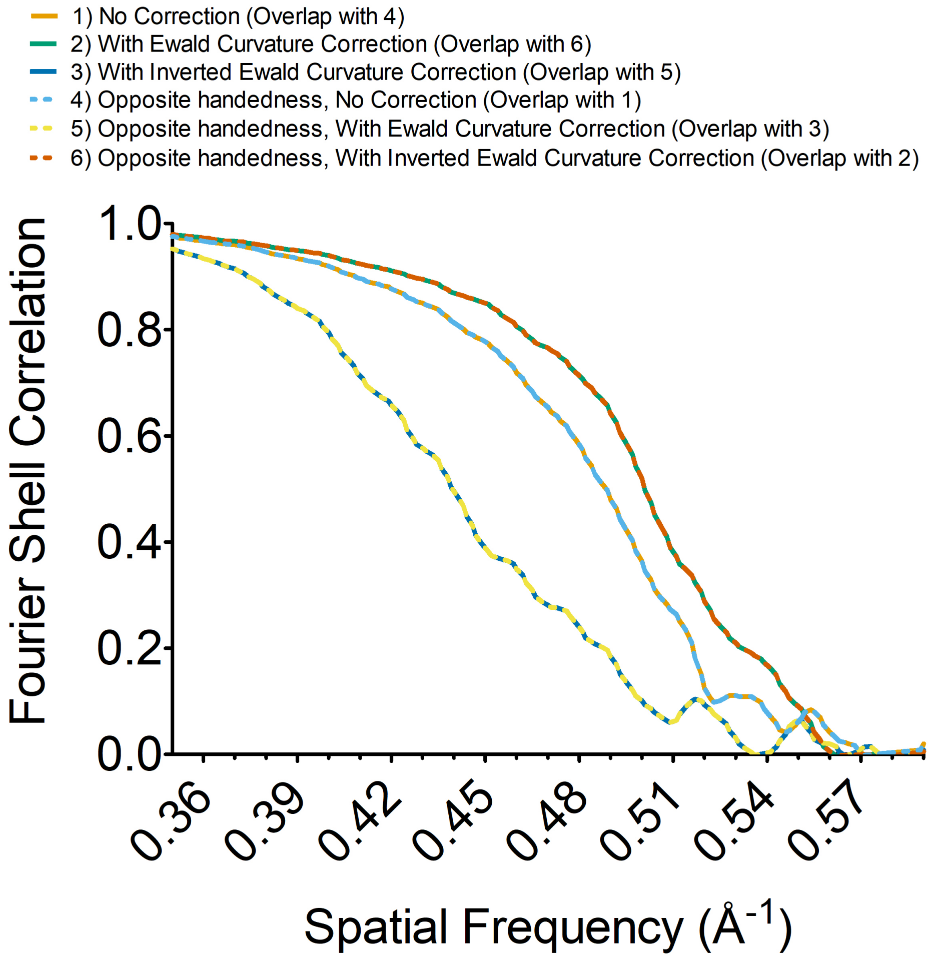

Supplementary Figure 7 | Examining correlation of defocus with resolution for reconstructions of $AAV2_{L336C}$. Outlier particles with a Frealign score below 4 and defocus above 2 μm were removed from this analysis. (**a**) Scatter plot of Frealign scores against average defoci for the data, with ranges between 0.35-1.0 μm and 1.0-2.0 μm. (**b**) Histogram of the average defoci of all particles. (**c**) FSC curves of reconstructions from each of the half-sets. Both datasets show similar curves. (**d**) Histogram of the average defoci of 5000 particles with the smallest (orange) and greatest (blue) defoci. (**e**) FSC curves for reconstructions from each of the 5000 particles from panel (d) are nearly identical. (**f**) FSC curves show that using a smaller box size does not change the resolution for the reconstruction from particles closer to focus, but (**g**) results in a lower resolution reconstruction from particles further from focus, due to aliasing effects. Defocus range for panels (f) and (g) and the coloring scheme for the different box sizes are described within the panel.

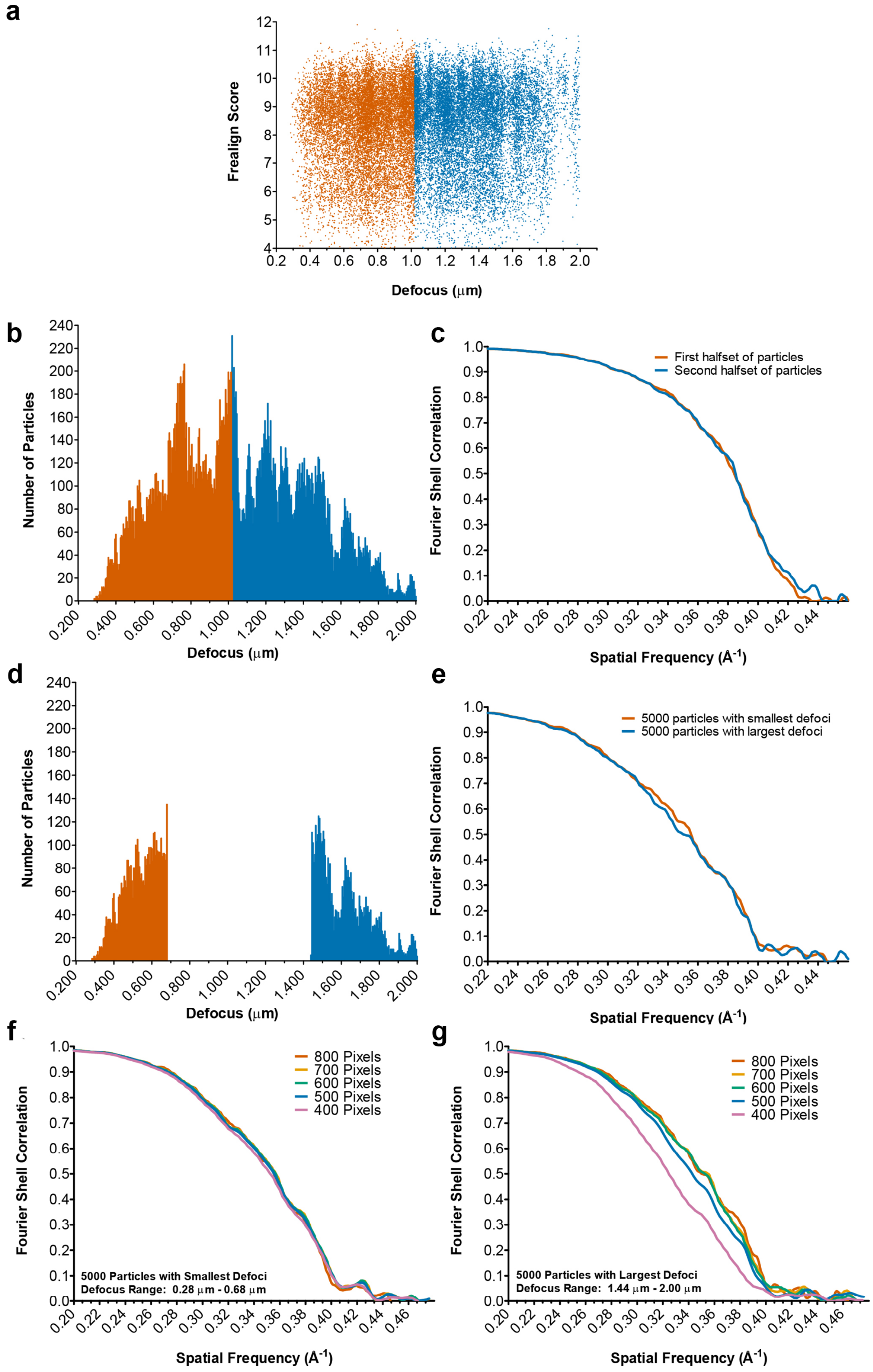
a
Frealign Score
Defocus (μm)
b
Number of Particles
Defocus (μm)
c
Fourier Shell Correlation
First halfset of particles
Second halfset of particles
Spatial Frequency (Å^{-1})
d
Number of Particles
Defocus (μm)
e
Fourier Shell Correlation
5000 particles with smallest defoci
5000 particles with largest defoci
Spatial Frequency (Å^{-1})
f
Fourier Shell Correlation
800 Pixels
700 Pixels
600 Pixels
500 Pixels
400 Pixels
5000 Particles with Smallest Defoci
Defocus Range: 0.28 μm - 0.68 μm
Spatial Frequency (Å^{-1})
g
Fourier Shell Correlation
800 Pixels
700 Pixels
600 Pixels
500 Pixels
400 Pixels
5000 Particles with Largest Defoci
Defocus Range: 1.44 μm - 2.00 μm
Spatial Frequency (Å^{-1})

Supplementary Figure 8 | Delineation of H-atom contributions within the AAV_{L336C} density map. Representative examples of amino acid side chains that begin to show density associated with hydrogen atoms are depicted in this figure. While we cannot yet distinguish individual hydrogen atoms, their indications are becoming evident within the maps. The coloring scheme for atoms is the same as in Figure 3. Residues shown in this figure are Y275 (top left), P481 (top right), W606 (bottom left) and F637 (bottom right).

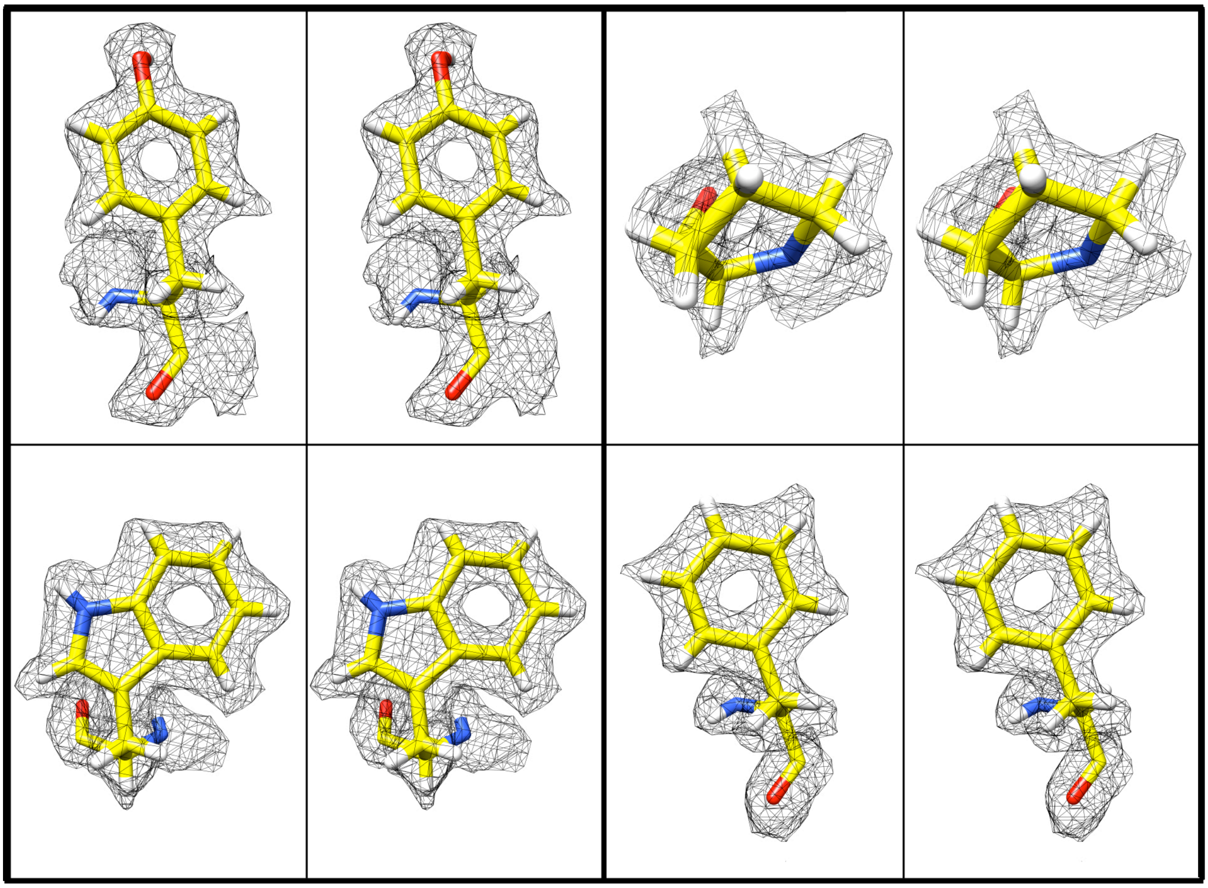

Supplementary Figure 9 | Improvement of a previously published dataset with per-particle CTF estimation. Resolution curves describing reconstructions of the 60S ribosomal subunit from Passos *et al.*[4] (green) and from this work, after applying per-particle CTF estimation (red). At these resolutions, correcting for the curvature of the Ewald sphere did not significantly improve the map, in line with our observations in Figure 2.

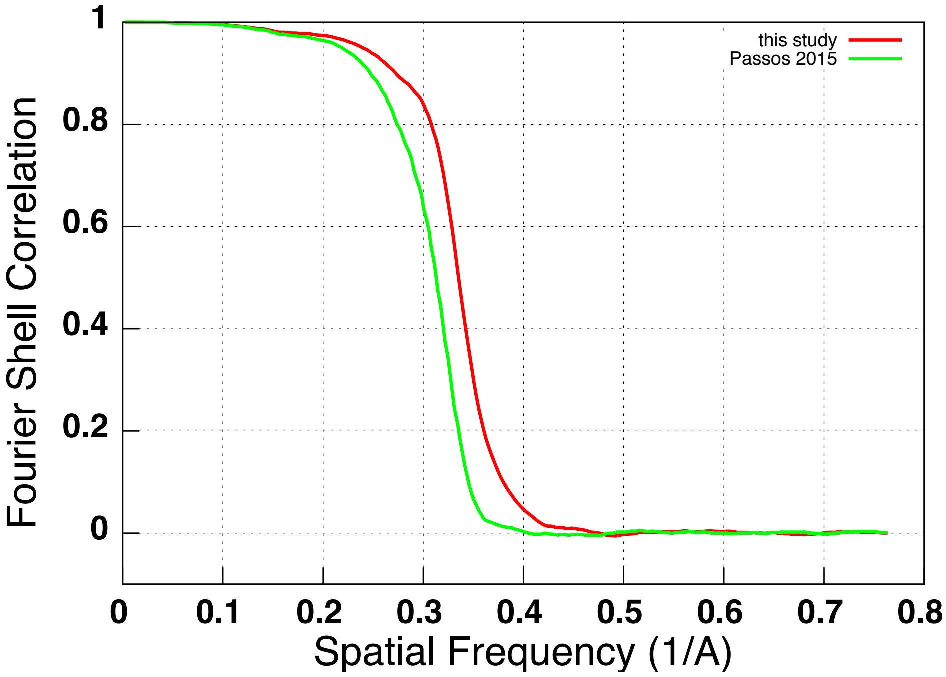

Supplementary References

1. Lander, G.C. et al. Appion: an integrated, database-driven pipeline to facilitate EM image processing. *J Struct Biol* **166**, 95-102 (2009).
2. Wolf, M., DeRosier, D.J. & Grigorieff, N. Ewald sphere correction for single-particle electron microscopy. *Ultramicroscopy* **106**, 376-382 (2006).
3. Russo, C.J. & Henderson, R. Ewald sphere correction using a single side-band image processing algorithm. *Ultramicroscopy* (2018).
4. Passos, D.O. & Lyumkis, D. Single-particle cryoEM analysis at near-atomic resolution from several thousand asymmetric subunits. *Journal of structural biology* **192**, 235-244 (2015).
