# Supplementary material for: Sub-2 Å Ewald curvature corrected structure of an AAV2 capsid variant
Source: Nat Commun. 2018 Sep 7;9:3628. doi: 10.1038/s41467-018-06076-6 (PMC6128836; doi:10.1038/s41467-018-06076-6)
Supplement: Supplementary file 2 — Editorial Note [file 41467_2018_6076_MOESM2_ESM.pdf]

Editorial Note: this manuscript has been previously reviewed at another journal that is not operating a transparent peer review scheme. This document only contains reviewer comments and rebuttal letters for versions considered at Nature Communications.
