# Supplementary material for: Sub-2 Å Ewald curvature corrected structure of an AAV2 capsid variant
Source: Nat Commun. 2018 Sep 7;9:3628. doi: 10.1038/s41467-018-06076-6 (PMC6128836; doi:10.1038/s41467-018-06076-6)
Supplement: Supplementary file 3 — Description of Additional Supplementary Files [file 41467_2018_6076_MOESM3_ESM.pdf]

Description of Additional Supplementary Files

File Name: Supplementary Movie 1
Description: Slice through the density map and model
